# Oral Lichen Planus in Thai Patients Has a Low Prevalence of Human Papillomavirus

**DOI:** 10.1155/2013/362750

**Published:** 2013-05-26

**Authors:** Pratanporn Arirachakaran, Jira Chansaengroj, Woradee Lurchachaiwong, Patnarin Kanjanabud, Kobkan Thongprasom, Yong Poovorawan

**Affiliations:** ^1^Department of Oral Medicine, Faculty of Dentistry, Chulalongkorn University, Bangkok 10330, Thailand; ^2^Oral Diseases Unit, Faculty of Dentistry, Chulalongkorn University, Bangkok 10330, Thailand; ^3^Center of Excellence in Clinical Virology, Faculty of Medicine, Chulalongkorn University, Bangkok 10330, Thailand

## Abstract

*Background*. Oral lichen planus (OLP) is a common chronic inflammatory immune-mediated disease, with an etiopathogenesis associated with cell-mediated immunological dysfunction. Viral infection has been hypothesized as a predisposing factor in the pathogenesis of this disease. Viruses may alter host cell function by inducing the abnormal expression of cellular proteins leading to disease development. However, reports on the relationship between human papillomavirus (HPV) and OLP are inconclusive. *Objective*. To explore the association between HPV and OLP in Thai patients. *Materials and Methods*. DNA was extracted from thirty-seven fresh-frozen tissue biopsy specimens from OLP lesions, and polymerase chain reaction assay for the L1 and E1 genes covering 32 types of high- and low-risk HPV was performed. *Results*. HPV DNA was detected in one tissue biopsy from an atrophic-type OLP lesion. All control samples were negative. Genomic sequencing of the E1 gene PCR product demonstrated that the HPV-type 16 found in the lesion is closely related to the East Asian type. *Conclusion*. Our data indicate a low prevalence of HPV infection in OLP lesions in Thai patients.

## 1. Introduction

Oral lichen planus (OLP) is a chronic inflammatory disease in which the immunopathogenesis involves cell-mediated immune dysregulation [1]. OLP affects 0.5–2.2% of the global population. However, prevalence varies according to geographic location [2]. This disease is commonly seen in Thai patients [3], with presentations ranging from symptom-free to a burning sensation or severe pain interfering with phonation, mastication, and deglutition [1]. 

OLP is classified as a potentially malignant disorder (PMD) of the oral mucosa with a transforming rate of 0–6.25% [1, 4]. Histologically, lesions are characterized by hyperkeratosis, basal layer liquefaction of the oral epithelium-connective tissue interface, and a dense infiltration of a band of lymphocytes [5, 6]. Unidentified antigen presented to lymphocytic cells may play a role in this disease. Previous studies suggest that OLP is a T-cell-mediated inflammatory disease in which autocytotoxic CD8^+^T cells trigger apoptosis in oral epithelial cells [1]. Exogenous agents may also alter keratinocyte antigen expression. The response of these specific CD8^+^T cells is similar to what occurs during a viral infection where a virus can act as a cytoplasmic antigen or induce the expression of host cell proteins, resulting in an altered host cell protein profile [7]. Therefore, it is of interest to investigate the possibility of viral involvement in the pathogenesis of OLP.

Human papillomaviruses (HPVs) are epitheliotropic DNA viruses with more than 150 genotypes. Clinically, HPV infection is usually characterized by hyperplastic, papillomatous, or verrucous lesions in the stratified epithelium. HPV classification has been based on the degree of HPV DNA homology. HPV has been detected in various types of oral lesions, ranging from benign to malignant [8, 9]. HPV typing can be valuable as a predictor of disease progression. Approximately 40 types are known to infect the oral cavity and urogenital tract [10]. HPVs are divided into high and low oncogenic risk groups [11]. HPV types 2, 3, 6, 11, 13, 16, 18, 31, 33, 35, 45, 52, and 57 are classified as high-risk (HR) types and reported to be associated with malignancy [11]. Twenty-four low-risk (LR) HPV genotypes (HPV 1, 2, 3, 4, 6, 7, 10, 11, 13, 16, 18, 30, 31, 32, 33, 35, 45, 52, 55, 57, 59, 69, 72, and 73) are associated with benign lesions such as papilloma and condyloma [10, 12]. HPV16 and HPV18 have increasingly been reported as being associated with PMD, and oral squamous cell carcinoma (OSCC) [13–17]. A meta-analysis including data from the years 1966–2010 reported that in comparison with normal mucosa, HPV was detected 2-3 fold more frequently in PMD and OSCC [9].

A causal role of HPV has been reported for OLP and OSCC, but there are wide variations in disease prevalence with regard to different geographic populations [1, 18]. Furthermore, data obtained from the International biological study of cervical cancer (IBSCC) showed phylogenetic clusters of HPV-16 sequence variants in direct relation to specific racial groups and geographic locations [19]. 

Most HPV infections are asymptomatic; however, lesions can develop any time after infection. Currently, there is no clear understanding of HPV latency, reactivation, or subclinical infection without apparent disease [20]. The course of HPV infection is dependent on the subtype and associated cofactors such as smoking, nutritional status, immune status, and hormonal influence [8]. 

The aims of this study were to investigate the presence of HPV DNA in OLP lesions of Thai patients using PCR and to characterize the virus type in those lesions by nucleotide sequencing. 

## 2. Materials and Methods

### 2.1. Sample Collection

Participants with a clinical diagnosis of OLP who visited the Department of Oral Medicine, Faculty of Dentistry, Chulalongkorn University from November 2008 to August 2010 were recruited into the study. Prior to enrollment, the patients signed informed consent forms approved by the Ethics Committee of the Faculty of Dentistry. None of the participants received any medication for their OLP before specimens were obtained. Specimens were collected from tissue biopsies for histopathological examination. Thirty-seven cases that were confirmed as OLP positive based on histopathological evaluation were selected for DNA extraction. Each specimen was transferred to a 1.5 mL sterile microcentrifuge tube containing 0.5 mL of virus transport media. Matching controls from each subject were obtained by scraping healthy normal mucosa into a 1.5 mL sterile microcentrifuge tube containing virus transport media. All samples were kept on ice, immediately taken to the laboratory, and stored at −80°C until further analysis.

### 2.2. DNA Extraction, HPV Amplification and Sequencing

DNA extraction and purification from both tissue biopsies and negative control cells were performed using the Qiamp DNA mini kit (QIAGEN, Valencia, CA) per the manufacturer's instructions. DNA extraction from control sites was performed by standard organic extraction (phenol-chloroform) and alcohol precipitation. Purified DNA was re-suspended in deionized water to a final volume of 30 *μ*L.

 We assayed for HPV DNA using primers to both the E1 and L1 regions. The primer design, amplification, and sequencing were performed [21]. Briefly, the first round PCR was performed using HPV-E1F1_1219 (nt 1160–1183) sequences; 5′-AGTACAGGTTCTAAAACGAAAGT-3′ and HPVE1R1_2119 (nt 2076–2100) 5′-CATTATCAAATGCCCAYTGYACCAT-3′. These primers yield a PCR product of 940 bp. The second round PCR was performed using 1 *μ*L of product from the first round as template and HPV-E1F2_1383 (nt 1315–1335) sequences; 5′-GCGAAGACAGCGGNTATGGC-3′ and HPV-E1R1_2119 as primers. These primers yield a PCR product of 785 bp. The use of these consensus primers allowed the amplification of a broad spectrum of HPV-type DNA. The MY09/MY11 and GP5+/GP6+ primer sets are the most widely used in detecting the HPV L1 gene region. The house keeping *β*-globin gene was used as an internal control. The PCR products were subjected to 2% agarose gel (FMC Bio-products Rockland, ME) electrophoresis, and appropriately sized bands were purified using the HiYield Gel/PCR DNA Fragments Extraction kit (Bioscience). The purified DNA fragments were sequenced by FirstBASE Laboratories SDNBHD (Selangor Darul Ehsan, Malaysia) and were analyzed using the BLAST analysis tool in the NCBI database (http://blast.ncbi.nlm.nih.gov/Blast.cgi).

### 2.3. Phylogenetic Tree Construction

The sequences were aligned with the BioEdit program (version 7.0.4.1). The phylogenetic trees were constructed using the MEGA4 program with neighbor-joining analysis, and Bootstrapping applied with 1,000 replicates was used to support tree topologies. 

## 3. Results

**Figure 1:**
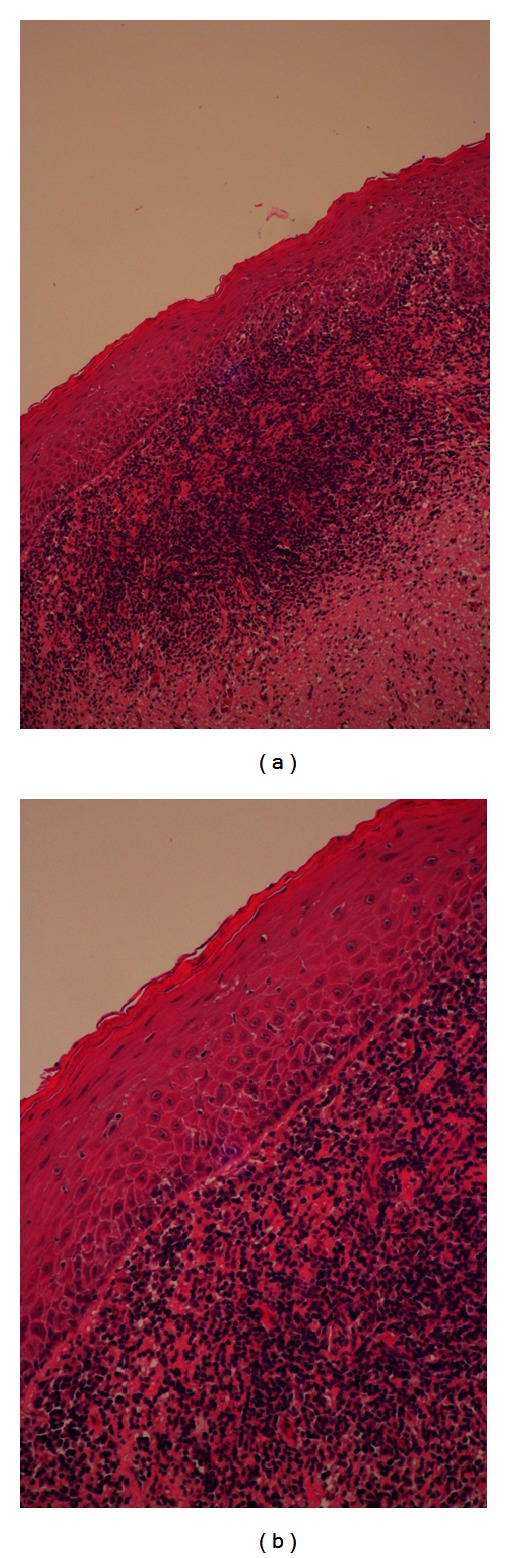
Histopathology of tissue biopsies confirming oral lichen planus. (a) Magnification ×100 shows hyperkeratosis, a bandlike of lymphocytes infiltrate in the superficial lamina propria, and (b) magnification ×200 shows degeneration of basal cell layer, various degrees of parakeratosis, and orthokeratosis of the surface epithelium, dense infiltrate of lymphocytes subjacent to the epithelium, and degenerating keratinocytes.

**Figure 2:**
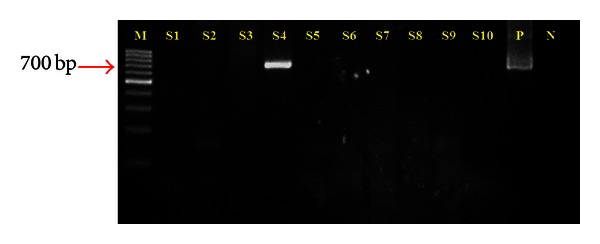
Agarose gel electrophoresis result of human papillomavirus E1 nested PCR detection. M: Marker, DNA ladder (Fermentas), S: sample, P: positive control, and N: negative control. DNA band in S4 indicates the sample with positive result.

**Figure 3:**
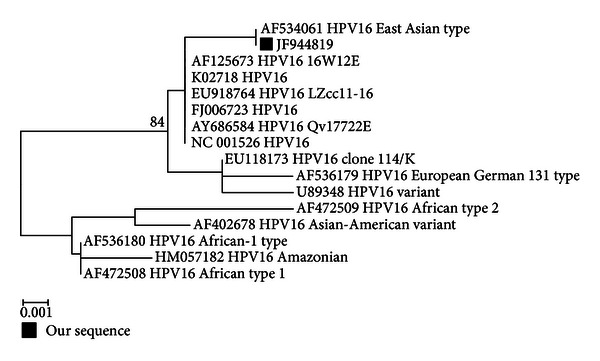
Phylogenetic tree in E1 region of HPV16 variants.

The study comprised 37 participants, ranging from 19–78 years old, with a median and mean age of 48 and 49 (±12.58) years, respectively. The male to female ratio was 12 : 25. The 37 specimens were obtained from 12 hyperplastic-, 23 atrophic-, and 2 ulcerative-type OLP lesions. All subjects had lesions on both the buccal mucosa and gingiva at the time of biopsy. All specimens from the tissue biopsies had a confirmed diagnosis of OLP (Figure 1). HPV DNA was detected in one of the specimens (2.7%), and the PCR result of the positive sample is shown in Figure 2. The HPV DNA positive sample came from an atrophic-type OLP lesion. None of the control samples obtained by scraping is tested positive for HPV DNA. The result of the BLAST analysis of the positive specimen showed a 100% homology to HPV16. The phylogenetic tree constructed with HPV 16 indicated that this virus was related to the East Asian variants (Figure 3). The accession number of the submitted sequence is JF944819. 

## 4. Discussion

OLP affects 1.27% of the global population with a prevalence varying according to geographic locations [2]. OLP predominantly affects middle-aged women between 30–60 years old [1]. In the present study, HPV DNA was found in an oral lesion of a 72-year-old female who presented to the oral medicine clinic complaining of a burning sensation in the oral cavity for one month prior to seeking treatment. Clinical examination revealed lesions suggestive of OLP on both the left and right buccal mucosa and her marginal gingiva. Biopsy was taken from the right buccal mucosa, and a histopathological evaluation confirmed the diagnosis of OLP and excluding malignancy. 

The present study supports the finding of a previous report on HPV prevalence in OLP in Thai patients. A prior study in Thai patients did not find HPV in any of the sixteen OLP lesions examined [22], while in the current study HPV was detected in only one of the 37 fresh tissue biopsies, indicating a low prevalence of 2.7%. Due to the very low prevalence of HPV in OLP lesions found in the present study, we were limited in our ability to analyze confounding risk factors such as age, sex, sexual habits, history of smoking, and alcohol consuming for OLP. 

A systematic review shows a statistically significant twofold difference (11% versus 23%) between HPV associated with OLP lesions and HPV found in normal tissue [9]. Among studies with positive results, there was a high degree of variation in experimental methods, ranging from differences in selection criteria and specimen collection, to methods of detection that affect the sensitivity and specificity of each study [9, 13–15, 18, 23–26]. 

The *in vitro* study of HPV is difficult since HPV cannot be grown using conventional cell culture methods, requiring other techniques. The choice of analysis technique used is also important. Kellokoski et al. [27] reported analyzing healthy oral mucosa for HPV DNA using dot blot hybridization, and polymerase chain reaction (PCR) resulted in positive findings in 3.8% and 29.4% of samples, respectively. PCR is considered to be a very sensitive detection method. In the present study, PCR was used to amplify and detect HPV DNA target sequences from fresh tissue samples by using consensus primers targeting conserved regions of the HPV genome. Such primers allow the amplification of a broad spectrum of HPV-type DNA. Although PCR amplification of the SPF10 (L1) gene is widely used in commercially available test kits, we also selected primers from the E1 gene, designed to cover 32 of the HPV DNA genotypes including HPV 6, 11, 16, 18 among other low- and high-risk HPVs (HPV 6, 11, 16, 18, 30, 31, 32, 33, 34, 35, 39, 40, 42, 44, 51, 52, 53, 55, 56, 58, 59, 66, 68, 70, 71, 73, 74, 81, 82, 85, 90, 91) [21]. PCR amplification with primers designed for early gene (E) regions is reported to be very sensitive and efficient [23, 28]. The E1 gene encodes functional proteins essential for viral replication and is a gene now used in a commercial product for classifying HPV genotypes (Papillo-Check, Frickenhausen, Germany). The E1 PCR assay can be used for HPV detection with a sensitivity of 10^2^ copies *μ*L^−1^ and also allows an accurate identification since it can amplify long nucleotide sequences [21]. 

Efforts in exploring the correlation between HPV and OLP have mainly focused on epidemiological studies of different populations. It is noteworthy that there have been profound variations in the results found among geographically different populations [1]. Studies in the USA using *in situ* hybridization on paraffin embedded specimens did not find any relationship between HPV and OLP [29, 30]. In contrast, patients with OLP in European countries are reported to have HPV prevalences ranging from 11.8% to 100% [9, 13, 15, 23–26]. Currently, twenty-four HPV types have been detected in oral PMD and OSCC. Notably, the most common type present in OSCC was HPV 16, at a prevalence of 16% [9, 17, 31, 32]. Previous reports on HPV prevalence in OLP showed HPV types 6, 11, 16, 18, 31, 32, 33, 39, or 55 present with the majority identified as type 6, 11, or 16 [9, 13, 14, 18, 24, 33]. A longitudinal study by Nielsen et al. [34] reported 40.8% of OLP lesions were HPV positive versus none in normal mucosa. The OLP cases that developed OSCC within 4–12 years were positive for HPV. 

The DNA sequence of the HPV found in the present study was identified as the high-risk-type 16. The lesion positive for HPV DNA was of the atrophic type. Mattila et al. [33] reported finding HPV DNA in 15.9% of atrophic OLP lesions. Moreover, 5 out of 13 atrophic OLP samples with HPV infection developed cancer. Among those five, three were infected by HR-HPV: types 16 and 33, and two were positive for types 6 and 11, which are LR-HPV. 

A predisposition of the oropharyngeal mucosa to malignant transformation by HPV was first suggested when HPV16 was detected in tumors of the tongue, tonsil, and pharynx but not in control tissues [35]. Furthermore, a large retrospective study of oropharyngeal cancer by Gillison et al. [36], indicated that poorly differentiated tumors were more likely to be HPV positive than well-differentiated and moderately differentiated tumors. A long-term followup is needed to determine if the aggressive behavior of HPV-infected OLP is associated with HR or LR type of HPV in malignant transformation. The HPV-type 16 sequence variation found from a Thai patient with OLP in the current study was phylogenetically classified as an East Asian type which originated from European variant [37]. Existing data on HPV sequence variation are mostly from studies of genital HPV [29] and oropharyngeal cancer [36]. Some investigations have proposed that HPV16 variants are associated with virus behavior including virus assembly, viral persistence, an increase in the risk of cancer invasiveness, and effect on the host immune response [19, 36, 38–41]. However, the distribution of the virus variants was also suggested to possibly be a simple reflection of the populations studied. Currently, data on sequence variation and characterization of HPV behavior from infected oral tissues is still very limited. Sequencing HPV DNA may be a valuable tool in predicting the aggressiveness of a premalignant lesion. Further studies that aimed at exploring the putative link between geographic location and HPV in OLP may reveal interesting findings. A prospective study of OLP with and without HPV DNA may show differences in disease progression of this potentially malignant disorder. 

## 5. Conclusion

OLP is an inflammatory immune-mediated disease associated with cell-mediated immunological dysfunction. Infectious agents have been proposed as one of the causes of OLP. Our data showed a low prevalence of HPV infection in OLP lesions. We conclude that HPV may not play a significant role in Thai patients with OLP. Genomic sequencing of the E1 gene demonstrated that the HPV-type 16 from the sole positive lesion was closely related to the East Asian type. Although it is likely that additional factors affect malignant transformation, identifying these will require the long-term followup of HPV-positive OLP patients.
